# ICE*Sp1109*, a Novel Hybrid Integrative Conjugative Element of Macrolide-Resistant *Streptococcus pyogenes* Serotype M77 Collected Between 2003 and 2017 in Poland

**DOI:** 10.1093/infdis/jiae473

**Published:** 2024-10-12

**Authors:** Jan Gawor, Karolina Żuchniewicz, Matthew Ojeda Saavedra, Stephen B Beres, Marlena Kiedrowska, Izabela Wróbel-Pawelczyk, Aleksandra Kozińska, Robert Gromadka, James M Musser, Izabela Sitkiewicz, Izabela Kern-Zdanowicz

**Affiliations:** Institute of Biochemistry and Biophysics, Polish Academy of Sciences, Warsaw, Poland; Institute of Biochemistry and Biophysics, Polish Academy of Sciences, Warsaw, Poland; Laboratory of Molecular and Translational Human Infectious Disease Research, Center for Infectious Diseases, Department of Pathology and Genomic Medicine, Houston Methodist Research Institute and Houston Methodist Hospital, Houston, Texas, USA; Laboratory of Molecular and Translational Human Infectious Disease Research, Center for Infectious Diseases, Department of Pathology and Genomic Medicine, Houston Methodist Research Institute and Houston Methodist Hospital, Houston, Texas, USA; Department of Epidemiology and Clinical Microbiology, National Institute of Medicines; Department of Epidemiology and Clinical Microbiology, National Institute of Medicines; Department of Epidemiology and Clinical Microbiology, National Institute of Medicines; Institute of Biochemistry and Biophysics, Polish Academy of Sciences, Warsaw, Poland; Laboratory of Molecular and Translational Human Infectious Disease Research, Center for Infectious Diseases, Department of Pathology and Genomic Medicine, Houston Methodist Research Institute and Houston Methodist Hospital, Houston, Texas, USA; Department of Biochemistry and Microbiology, Warsaw University of Life Sciences, Warsaw, Poland; Institute of Biochemistry and Biophysics, Polish Academy of Sciences, Warsaw, Poland

**Keywords:** *Streptococcus pyogenes*, macrolide resistance, M77/ST63, integrative conjugative element, genome sequencing

## Abstract

**Background:**

The antibiotic resistance determinants and associated mobile genetic elements (MGEs) were detected among *Streptococcus pyogenes* (group A streptococci [GAS]) clinical isolates of an M77 serotype collected in Poland between 2003 and 2017.

**Methods:**

The genomes of 136 M77 GAS isolates were sequenced using short- and selected with long-read approach; whole genome sequences were analyzed to determine the genetic context of macrolide resistance determinants.

**Results:**

The analysed strains were collected from in- and outpatients. Sequencing data analysis revealed that all strains carried the *tet*(O) gene. They were classified as a single sequence type, ST63. The unique erythromycin-resistance determinant, the *erm*(TR), was detected in 76.5% (n = 104) of isolates. It was found predominantly (n = 74) within a novel hybrid integrative conjugative element composed of the ICE*Sp1108*-like sequence and ICE*Sp2906* variant, which was then named ICE*Sp1109*. However, in strains isolated before 2008, *erm*(TR) was located within ICE*Sp2905* (n = 27) and in 3 strains - within stand-alone ICE*Sp1108*-like sequences.

**Conclusions:**

Based on phylogenetic analysis results, the clonal dissemination of the macrolide-resistant *S. pyogenes* M77/ST63 strain with hybrid ICE*Sp1109* was observed between 2008 and 2017. ICE*Sp1109* is the novel hybrid ICE in gram-positive bacteria.

## Teaser

A novel hybrid 103-kb integrative and conjugative element, ICE*Sp1109*, was identified in macrolide- and tetracycline-resistant *Streptococcus pyogenes* M77/ST63 strains collected in Poland between 2003 and 2017. Whole genome-based phylogenetic analysis revealed its clonal dissemination since 2008.

*Streptococcus pyogenes* (group A *Streptococcus* [GAS]) is a major human pathogen that causes a wide range of infections—from mild, such as pharyngitis or impetigo, to severe and invasive, such as septicemia, necrotizing fasciitis, and streptococcal toxic shock syndrome (for a review, see [1]). Although all GAS strains remain sensitive to β-lactams, and this class of antibiotics is a drug of choice to treat streptococcal infections, macrolides are recommended for patients allergic to β-lactams. Moreover, macrolide combined with β-lactams is preferred in treating severe or complicated *S. pyogenes* infections [2].

Resistance to macrolides in *S. pyogenes* primarily involves target site modification and depends on the presence of the *erm* genes, which code for a methylase of the ribosomal RNA (23S rRNA). The resulting modification of the ribosome prevents the binding of macrolide antibiotics. The *erm* genes confer resistance not only to macrolides, but also to lincosamides and streptogramin B; therefore, the phenotype presented by the bacteria carrying *erm* genes is called MLS_B_ (from macrolides, lincosamides, streptogramin B). The expression of the *erm* genes is predominantly inducible (iMLS_B_); however, the gene can be expressed constitutively (cMLS_B_) [3]. In *S. pyogenes*, few groups of the *erm* genes have been detected so far—*erm*(A), with its subtype *erm*(TR), *erm*(B), and *erm*(T). The second resistance mechanism relies on the concomitant action of an efflux pump, which exports macrolide antibiotics out of the bacterial cell, and a protein, which protects the ribosome by driving dissociation of bound macrolide from the ribosome. The associated phenotype, called the M phenotype, is characterized by low-level resistance to 14- and 15-membered macrolides (eg, erythromycin and clarithromycin). This type of resistance in GAS is specified by efflux pumps encoded by *mef*A or *mef*E genes, and an *msr*(D)-encoded ribosome protector [4].

Macrolide resistance genes in GAS are frequently located on integrative and conjugative elements (ICEs). ICEs are diverse mobile genetic structures, distributed in virtually all bacterial genera, integrated into chromosomes, that can excise, circularize, and transfer horizontally via conjugation to neighboring bacteria. Upon integration into bacterial chromosomes, ICEs generate directly repeated sequences (for a review, see [5]). ICEs are important players in bacterial evolution and the lateral spread of antibiotic resistance genes [6, 7].

In the last 2 decades, there has been a gradual increase in the prevalence of macrolide-resistant *S. pyogenes* (MRSP) isolates in Europe, with some countries rising to over 30%; Italy, Spain, and Greece are among the most affected. Recently, the decline of MRSP isolates has been observed in these countries [8–10]. In multiple studies conducted in different parts of the world, clonal proliferation of certain serotypes of GAS, such as M4, M28, M75, and M77, has been observed [8, 10–12]. One of the main drivers of macrolide resistance in *S. pyogenes* is the widespread use of macrolide antibiotics for the treatment of respiratory tract infections. The overuse and misuse of antibiotics have led to the emergence and spread of resistant strains, making treatment more difficult and expensive. Continued surveillance and monitoring of MRSP isolates are essential to prevent the spread of resistance and to ensure effective treatment of *S. pyogenes* infections. In Poland, the level of GAS resistance to erythromycin is estimated in the range of 12%, with a tendency to systematic growth [13]. Recent data estimate the prevalence of resistant strains at around 16%–18% [14]. Although the incidences of *S. pyogenes* infections are monitored, the data from Central Europe on the strain analysis are missing, so we hope to fill this gap. Here, we present the genomic analysis of MRSP clones of the M77 serotype collected in Poland between 2003 and 2017.

## MATERIALS AND METHODS

### *Streptococcus pyogenes* Strains

The 136 *S. pyogenes* M77 strains were collected between 2003 and 2017 from patients in 2 multicenter surveys, bacterial invasive infections NETwork (BINet) and respiratory tract infections NETwork (Alexander/RESPI-net) [15, 16], concerning community-acquired bacterial infections in Poland: 117 were isolated from respiratory tract infections, 3 from skin infections, 1 from urogenital tract infection, and 15 isolates were invasive (with 12 collected from blood; Supplementary File 1). A general description of these strains is presented in Sitkiewicz et al [14] and included as Supplementary File 1.

### Whole Genome Sequencing

Genomic DNA of all isolates was sequenced using a short-read approach with Illumina technology (Illumina Inc). To confirm the structure of mobile genetic elements (MGEs) carrying antibiotic resistance genes, 8 representative strains were selected for the long-read sequencing using Oxford Nanopore technology to obtain complete physical maps. Detailed information regarding the DNA isolation and sequencing strategies are described in Supplementary File 2. Sequence reads obtained during this study were deposited in the Sequence Read Archive database under BioProject ID PRJNA1098028. Complete *S. pyogenes* M77 genomes were deposited in the National Center for Biotechnology Information GenBank database under accession numbers CP155734–CP155741.

### Bioinformatic Analysis

Sequence type (ST) and *emm* type were determined using SRST2 v.0.2.0 (https://github.com/katholt/srst2). Sequence annotation was performed using DFAST v.1.2.18 (https://github.com/nigyta/dfast_core). Antimicrobial resistance genes and virulence genes were identified using Abricate v.1.0.0 (https://github.com/tseemann/abricate), based on the Comprehensive Antibiotic Resistance Database (CARD) [17] and Virulence Factor Database (VFDB) [18], respectively, retaining only hits with >90% identity and >80% target coverage. Core single-nucleotide polymorphism (SNP) phylogeny was inferred using snippy v.4.6.0 (https://github.com/tseemann/snippy) including masking of the prophage regions, followed by recombination removal by Gubbins [19] and phylogenetic tree construction using FastTree v.2.1 (http://www.microbesonline.org/fasttree/) with a generalized time-reversible model. Pairwise SNPs were calculated using snp-dists v.0.8.2 (https://github.com/tseemann/snp-dists). The data on *S. pyogenes* M77/ST63 strains isolated in other countries available in public databases were included in the phylogenetic analysis (Supplementary File 3). MGEs were identified within scaffolded genomes using MobileElementFinder v.1.1.2 [20], ICEfinder [21], VRprofile2 [22], and ICEScreen (https://icescreen.migale.inrae.fr/) tools. Prophage sequence detection was conducted using DEPhT v1.2.2 (https://github.com/chg60/DEPhT) and geNomad v.1.7.5 (https://github.com/apcamargo/genomad); manual inspection applying BLASTn [23] was performed to verify the identification results. The phylogenetic tree and heatmap figures presented in this study were visualized in R v.4.3.3 environment using ggtree v.3.10.1 (https://github.com/YuLab-SMU/ggtree) and ComplexHeatmap v.2.18.0 (https://github.com/jokergoo/ComplexHeatmap) packages, respectively.

## RESULTS

### Strain Characteristics

**Figure 1.**
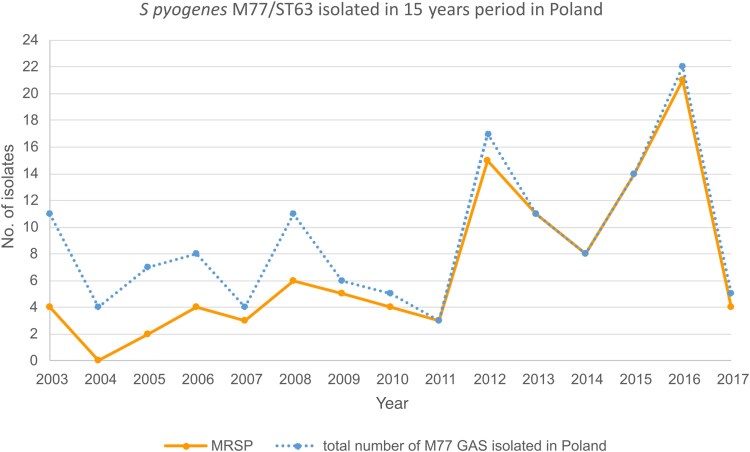
Temporal distribution of M77/ST63 macrolide-resistant *Streptococcus pyogenes* isolates in Poland between 2003 and 2017. Abbreviations: GAS, group A streptococci; MRSP, macrolide-resistant *Streptococcus pyogenes.*

All 136 GAS with the M77 serotype collected during the 2003–2017 period were tetracycline resistant; the majority of them (104 [76.5%]) were also erythromycin resistant. The macrolide resistance phenotype was assayed according to the European Committee on Antimicrobial Susceptibility Testing (http://www.eucast.org/clinical_breakpoints/); the double disk diffusion test was performed to distinguish between iMLS_B_ and cMLS_B_ phenotypes as described by Sitkiewicz et al [14]. Of the erythromycin-resistant strains, 93 exhibited the iMLS_B_ phenotype, and 11 strains the cMLS_B_ phenotype [14]. No strains with the M phenotype were detected. The temporal distribution of M77 MRSP collected in Poland over 15 years is presented in Figure 1. The total number of M77 GAS, as well as the MRSP isolates, increased from 2011 till 2016; however, we observed a dramatic drop in the number of isolates in 2017, despite a whole year’s collection of strains. This trend cannot be confirmed, as the M77 GAS strains’ temporal distribution was not analyzed after 2017.

### Whole Genome Sequencing Analysis

**Figure 2.**
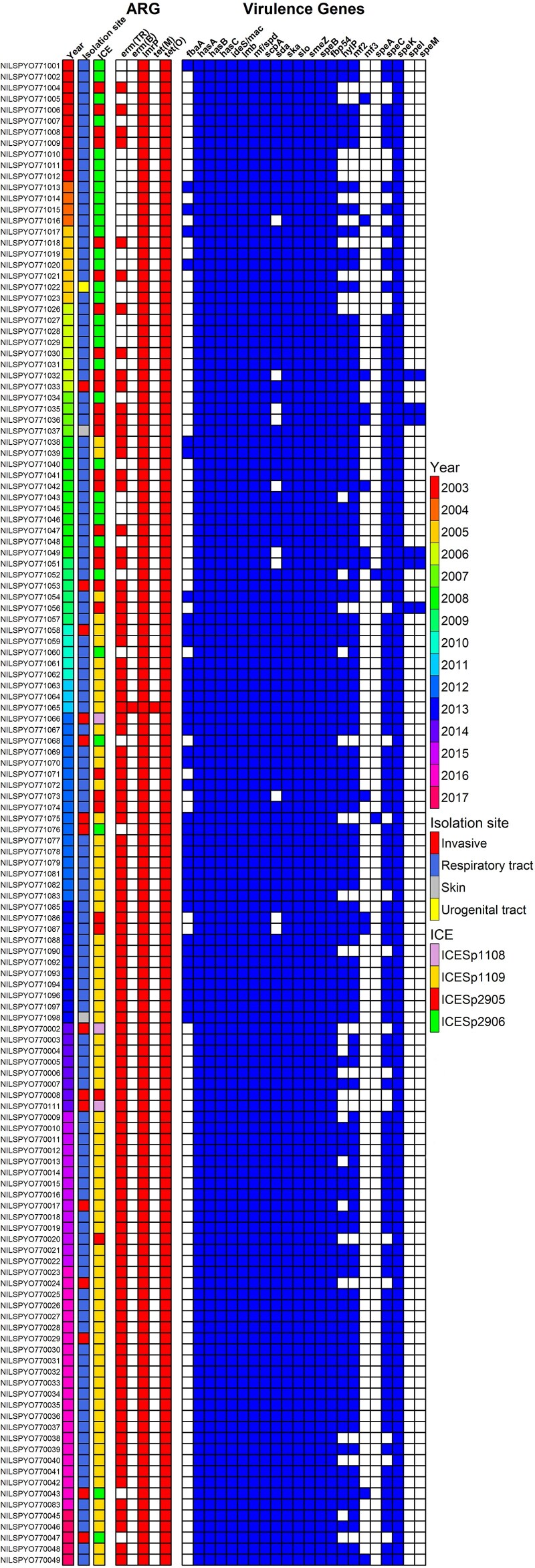
Presence/absence matrix depicting the antibiotic resistance (ARG) and virulence gene repertoire detected in *Streptococcus pyogenes* M77 isolates. The colored squares in the first, second, and third columns from the left represent the year of sample isolation, the strain isolation site, and the type of *erm*(TR) containing integrative and conjugative elements (ICE), respectively. The shaded squares in ARG heatmap column represent the presence of resistance determinants of macrolides [*erm*(TR), *erm*(B), *lmr*P] and tetracyclines [*tet*(M) and *tet*(O)] and the shaded squares in virulence genes column represent a presence/absence matrix of virulence factors.

All 136 strains were analyzed by the short-read whole genome sequencing (WGS) approach, and the 8 representative strains bearing different classes of ICEs carrying antibiotic resistance genes were subjected to long-read sequencing to confirm the correct assembly of those elements. The resulting genomic characteristics are presented in Supplementary File 1. The assembly utilizing long reads is also beneficial in proper genome assembly as the streptococcal chromosomes contain multiple insertion sequences (ISs) [24], which may affect assembly quality when only short reads are used. Genome alignment of *S. pyogenes*’ complete chromosomes obtained in this study reveals a high degree of synteny and a high level of nucleotide identity to the complete sequence of *S. pyogenes* NCTC13742 strain (Supplementary Figure 1). The summary of analyzed traits, such as year and anatomical site of isolation, presence of antibiotic resistance genes, and ICE content related to the macrolide resistance is presented in Figure 2.

All of the M77 strains represented ST63. Previously, the M77/ST63 isolates were also reported in Spain [10], Greece [8], the United Kingdom (UK) [25], and Iceland [26] as well as in other European countries [27, 28] and the United States (US), Australia, New Zealand, and Canada [28].

### Antibiotic Resistance Genes

The tetracycline determinant detected in all strains was the *tet*(O) gene (Figure 2), coding for the ribosome protection protein [29]. It is located on an ICE that has 99% nucleotide identity to ICE*Sp2906* described previously for the *S. pyogenes* strains isolated in Italy [6], except for the 6950-bps segment detected in Polish isolates inserted within *orf6* of ICE*Sp2906* (see further description in “*tet*(O)- and *erm*(TR)-Containing Elements section”). A single strain, NILSPYO771065, was found to harbor, in addition to *tet*(O), also *tet*(M), encoding another type of a ribosome protection protein, located within Tn*3872* [30], the Tn*916* family member.

All MRSP strains carried *erm*(TR) as the macrolide resistance determinant, and a single isolate, NILSPYO771065, contained additionally *erm*(B). The *lrm*P gene, coding for a proton motive force–dependent drug transporter [31], was detected in 136 genomes, both in macrolide-sensitive and -resistant strains. Therefore, we assume it did not confer macrolide resistance in vivo in the analyzed strains (Figure 2). The *mef*A, *mef*E, and *msr*(D) genes were not identified.

Most erythromycin-resistant strains were isolated between 2008 and 2017 (also considered newer strains in the analysis), and are classified predominantly to clade I based on core SNP analysis (Figure 5). Strains isolated before 2008 are either resistant or sensitive to erythromycin belonging mostly to clade II (older strains) (Figures 2 and  5).

### Virulence Genes

Detected GAS virulence factors (VFs) encoded in the core genome and on prophages are summarized in Figure 2. The number of VF genes encoded by a single isolate varies from 14 to 19, with most isolates (∼45%) carrying 17 genes. Thirteen VF genes, including *has*ABC coding for the capsule, have been identified in all 136 strains. The least frequent VF gene is *spe*A detected in 2 strains. The VF identification results are presented in detail in Supplementary File 4.

### Mobile Genetic Elements

#### Insertion Sequences, Prophages, and Other MGEs

Sequenced M77 GAS strains are similar to other sequenced strains of other GAS serotypes in terms of MGE content. In the analyzed M77 isolates, multiple ISs were identified—IS*110*, IS*21*, IS*256*, IS*3*, IS*30*, IS*As1*, and IS*L3* (Supplementary File 5)—all of which are common in streptococcal genomes [32]. The IS element repertoire identified in scaffolded genomes was validated by analyzing the complete chromosomes of 8 representative strains. The set of ISs detected was consistent across both scaffolded and complete genomes; however, the copy number of 2 ISs varied. An additional IS*As1* copy was found in all complete genomes, while an extra IS*3* copy was observed in 2 genomes (Supplementary File 5). These differences were due to the improved resolution provided by the assembly of complete chromosomes utilizing long sequencing reads.

We also identified 2 transposable elements—Tn*GBS2.3* and Tn*3872* [30, 33] (Supplementary File 6). Tn*GBS2.3* was identified in group B streptococci (GBS), but it was demonstrated to transfer to GAS via conjugation with relatively high frequency. Tn*3872*, described first in *Streptococcus pneumoniae*, belongs to the Tn*916-*family transposons reported initially in *Enterococcus faecalis*, constituting a large family of ICEs, common in Firmicutes (for a review, see [34]), that can harbor multiple genes conferring antibiotic resistance.

Interestingly, 96 M77 strains carry RD2 elements identified previously in *S. pyogenes* MGAS6180 and MGAS10270 [35]. This ICE can be transferred via conjugation to multiple GAS serotypes, and to GBS [36]. The RD2 element encodes numerous VFs and is considered the major element affecting the colonization potential of GAS strains [37], as it encodes cell surface–anchored adhesins and R28 protein.

**Figure 3.**
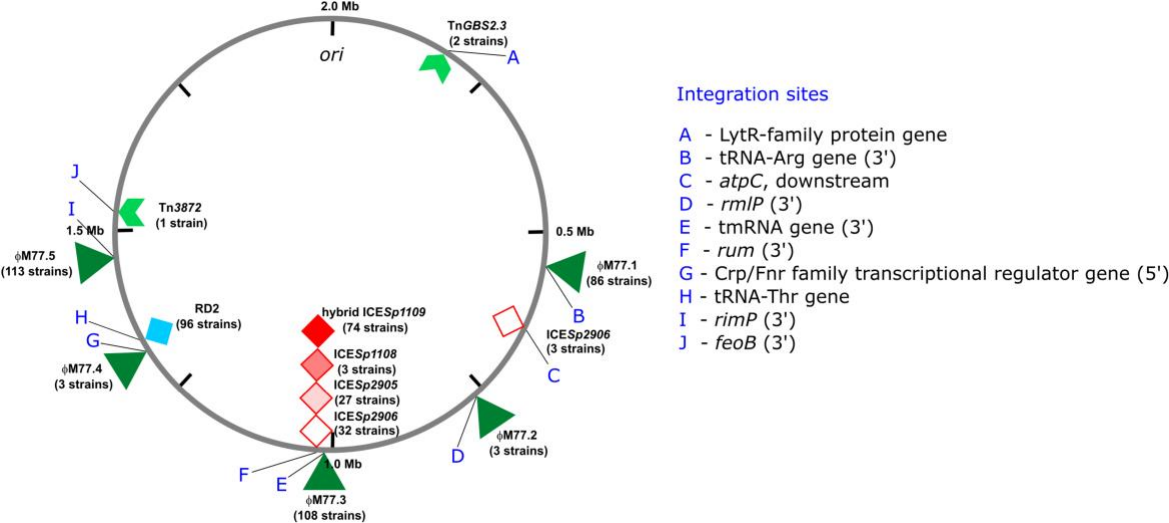
Schematic overview of the Polish *Streptococcus pyogenes* M77/ST63 core chromosome, prophage element insertion sites, and integrative and conjugative elements (ICEs). The circle represents the group A streptococci (GAS) chromosome with the marked nucleotide positions and the origin of replication (*ori*). The prophage elements are indicated with triangles and ICEs as squares that are shade-coded according to the ICE type; stacked squares indicate ICEs inserted at the same site. Letters denote the integration sites, described in the right panel. The presence of the given element in the sequenced GAS genomes is indicated.

We detected 5 prophages integrated into M77 genomes, namely ϕM77.1 to ϕM77.5 (Figure 3). Their nucleotide sequences are 96%–100% identical to known streptococcal phages. Phages ϕM77.1 and ϕM77.3 may be more common for M77 strains, while phage ϕM77.5 is widely spread in multiple GAS serotypes such as M1 or M12 and M77 (detailed analysis in Supplementary Files 7 and 8).

### *tet*(O)*-* and *erm*(TR)-Containing Elements

#### ICE*Sp2905* and Its Variants

**Figure 4.**
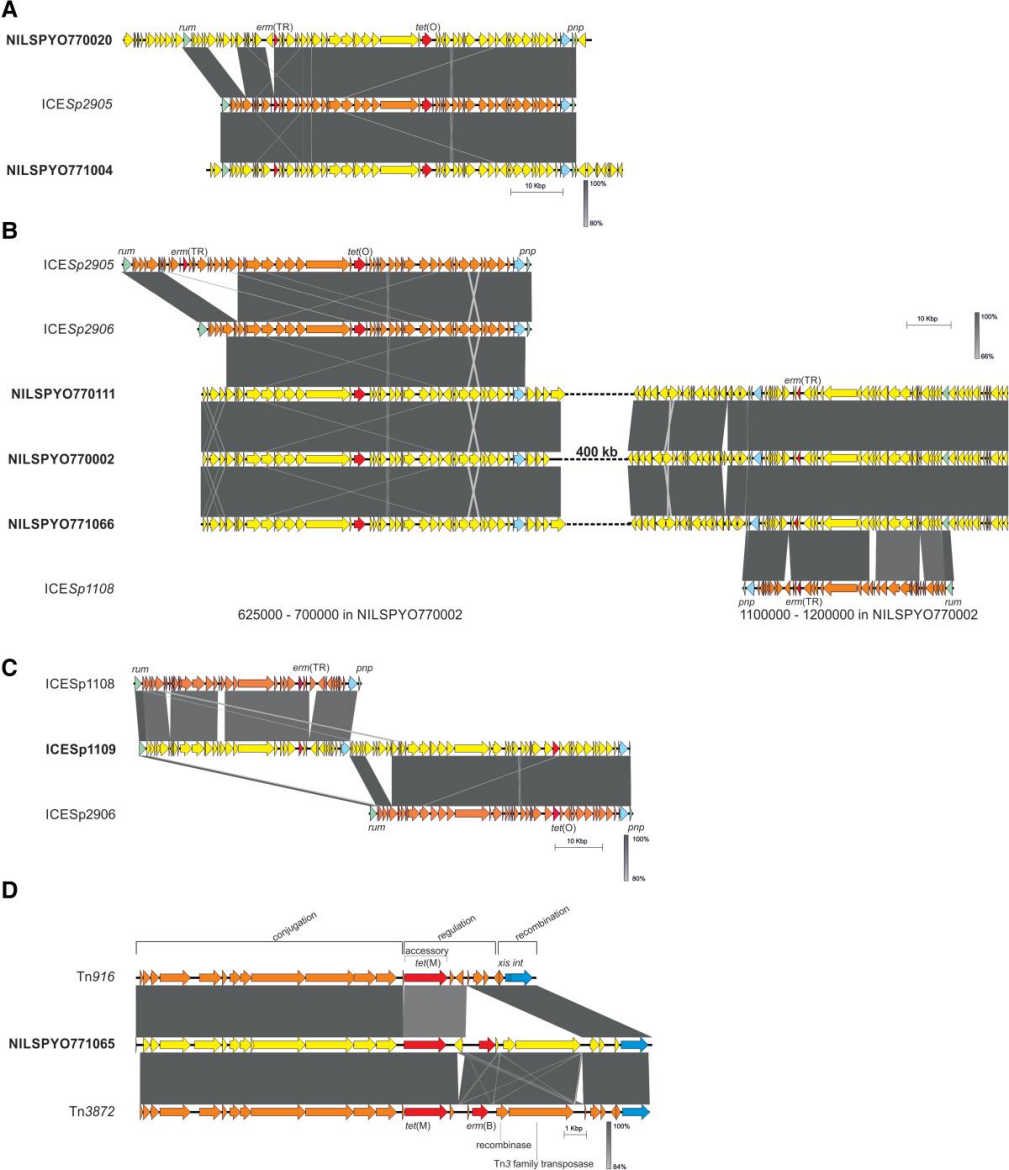
Schematic representation of the *erm*(TR)-containing integrative and conjugative elements (ICEs) identified in the genomes of *Streptococcus pyogenes* M77*/*ST63 strains. *A*, ICE*Sp2905* in the NILSPYO771004 genome and the ICE*Sp2905* variant in the NILSPYO770020 genome. *B*, The *tet*(O)*-* and *erm*(TR)-containing ICEs in the NILSPYO770002, NILSPYO770111, and NILSPYO771066 genomes. The genomic location of the specific fragment is marked. *C*, ICE*Sp1109*, the novel *erm*(TR)*–tet*(O)*-*containing hybrid element present in the NILSPYO771038 strain. *D*, Tn*3872* of the NILSPYO1065 strain, comprising the *tet*(M) and *erm*(B) genes. Tn*3872* (GenBank accession number OP715845.1) and Tn*916* (GenBank accession number U09422) are shown. The functional modules of Tn*916* are marked: conjugation, regulation, recombination, and the accessory gene *tet*(M). Antibiotic resistance genes as well as the *rum* and *pnp* genes are marked as shaded arrows.

The *tet*(O)-containing element ICE*Sp2906* [6] was detected in all collected M77/ST63 strains. It was either alone, (n = 34; in macrolide-sensitive strains n = 32), or as a hybrid element with IME*Sp2907* (n = 27), an integrative mobilizable element, constituting ICE*Sp2905* [6] (Figure 4*A*). ICE*Sp2906* was also detected as a hybrid with an ICE*Sp1108*-like element (n = 75) forming a novel element, ICE*Sp1109* (see below). Both ICE*Sp2905* and ICE*Sp1108* [7] are integrated into the M77/ST63 strain genomes between the *rum* and *pnp* genes, coding for the 23S rRNA m(5)U(1939)methyltransferase and a phosphorylase superfamily protein [6], respectively. Such integration is catalyzed by a site-specific serine integrase, encoded by a gene located at the 3′ ICE proximity [38].

In MRSP isolates, IME*Sp2907* was the source of the *erm*(TR) gene, similar to what was detected in *S. pyogenes* in Italy [6, 7]. In 3 strains (NILSPYO770002, NILSPYO770111, and NILSPYO771066), besides ICE*Sp2906* another ICE bearing *erm*(TR) was detected, with sequence blocks identical in 94%–100% to ICE*Sp1108*, and the 2 mentioned ICEs were located in the genome approximately 400 kbps apart from each other (Figure 4*B*). It should be stressed that compared to the original ICE*Sp2906* sequence [6], the 5′ terminus of ICE*Sp2906* of the 3 mentioned strains contains an insertion of 6950-bp sequence comprising 6 additional *orf*s. The extended ICE*Sp2906* with such an insertion was identified in all Polish *S. pyogenes* M77/ST63 strains. Moreover, a structure identical in 99% to the modified ICE*Sp2906* element was also detected in the *S. pyogenes* NCTC13742 (accession number LS483386.1) and *S. pyogenes* TSPY453 (accession number CP033337.1) genomes; these are also M77/ST63 strains and were isolated in 2015 in the UK and 2014 in the US, respectively. The origin of the entire 6950-bp fragment is unknown, although fragments identical in 86%–94% to some parts of this sequence (coverage 78%–92%) could be detected in various streptococci—*Streptococcus equi* subsp *zooepidemicus* (strains SEZ33, SEZ25, NCTC12090, and others), *Streptococcus dysgalactiae* subsp *equisimilis* 89, and *Streptococcus suis—*but also in the genomes of other bacteria related to the human microbiome such as *Filifactor alocis* ATCC 35896, or *Aerococcus* spp.

The ICE*Sp2905* element was identified in 27 isolates collected predominantly in the first years of the analyzed period. Interestingly, the canonical version of this element, identical (99%–100%) to the sequence deposited in the European Molecular Biology Laboratory database (accession number FR691055) [7], was detected in 12 strains while other strains carry the ICE*Sp2905* variants with an insertion within *orf6* of this element (Figure 4*A*). The localization and the composition of individual elements in the representative strains, NILSPYO771004 with ICE*Sp2905* and NILSPYO770020 with the ICE*Sp2905* variant, were confirmed by de novo assembling of their complete chromosomes.

It is worth noticing that in the case of 3 strains, NILSPYO770002 (complete genome assembly), NILSPYO770111 (scaffolded genome), and NILSPYO771066 (scaffolded genome), with distantly located ICE*Sp2906* and the ICE*Sp1108*-like elements, ICE*Sp2906* is deprived of its terminal sequences at the 5′ end, and only the terminal 43-bp *rum* fragment is present; the 3′ end is truncated by the 1055-bp DNA fragment including entire *pnp* together with terminal 36 bp of the preceding gene (Figure 4*B*).

### ICE*Sp1109*, a Novel Hybrid Element

In the majority of strains collected between 2008 and 2017 (n = 75), the ICE*Sp1108*-like element (94%–99% nucleotide identity) was inserted in the very 5′ flank of ICE*Sp2906*, giving rise to a novel hybrid element that we named ICE*Sp1109* (Figure 4*C*, Supplementary File 9). Similarly to ICE*Sp2905* and ICE*Sp1108*, ICE*Sp1109* integrated between the *rum* and *pnp* genes. The genomes of the first collected strains comprising ICE*Sp1109*, NILSPYO771038, and NILSPYO771039, and the last one, NILSPYO770049, were assembled into complete chromosomes. So far, the ICE*Sp1109* hybrid element has not been described nor deposited in available public databases. Direct repeats, also known as attachment sites left and right (*att*L and *att*R), generated upon ICE integration were detected in NILSPYO771038 for both ICE*Sp1109* components, ICE*Sp1108*-like and ICE*Sp2906* variant [7]. The putative sequences, *att*L and *att*R of the ICE*Sp1108*-like element, were identified within the 3′ end of the *rum* gene and in the serine recombinase gene, respectively (Supplementary Figure 2). The *att*L sequence of ICE*Sp2906* was detected to overlap *att*R of the ICE*Sp1108*-like element, and *att*R of ICE*Sp2906* within the ICE*Sp2906* serine recombinase gene (Supplementary Figure 2). The experimental data are required to determine whether ICE*Sp1109* is active as an entire element.

### Tn*3872*, a *tet*(M)*-* and *erm*(B)-Containing Element

In the genome of the single strain NILSPYO771065, besides the *erm*(TR) and *tet*(O) genes detected within ICE*Sp1109*, we also identified *erm*(B) and *tet*(M), located on Tn*3872* (Figure 4*D*), which is part of a larger element of the Tn*5252* superfamily, >62 kb in size (Supplementary Figure 3, detailed analysis in Supplementary File 10).

### Phylogenetic Analysis of M77/ST63 Strains

**Figure 5.**
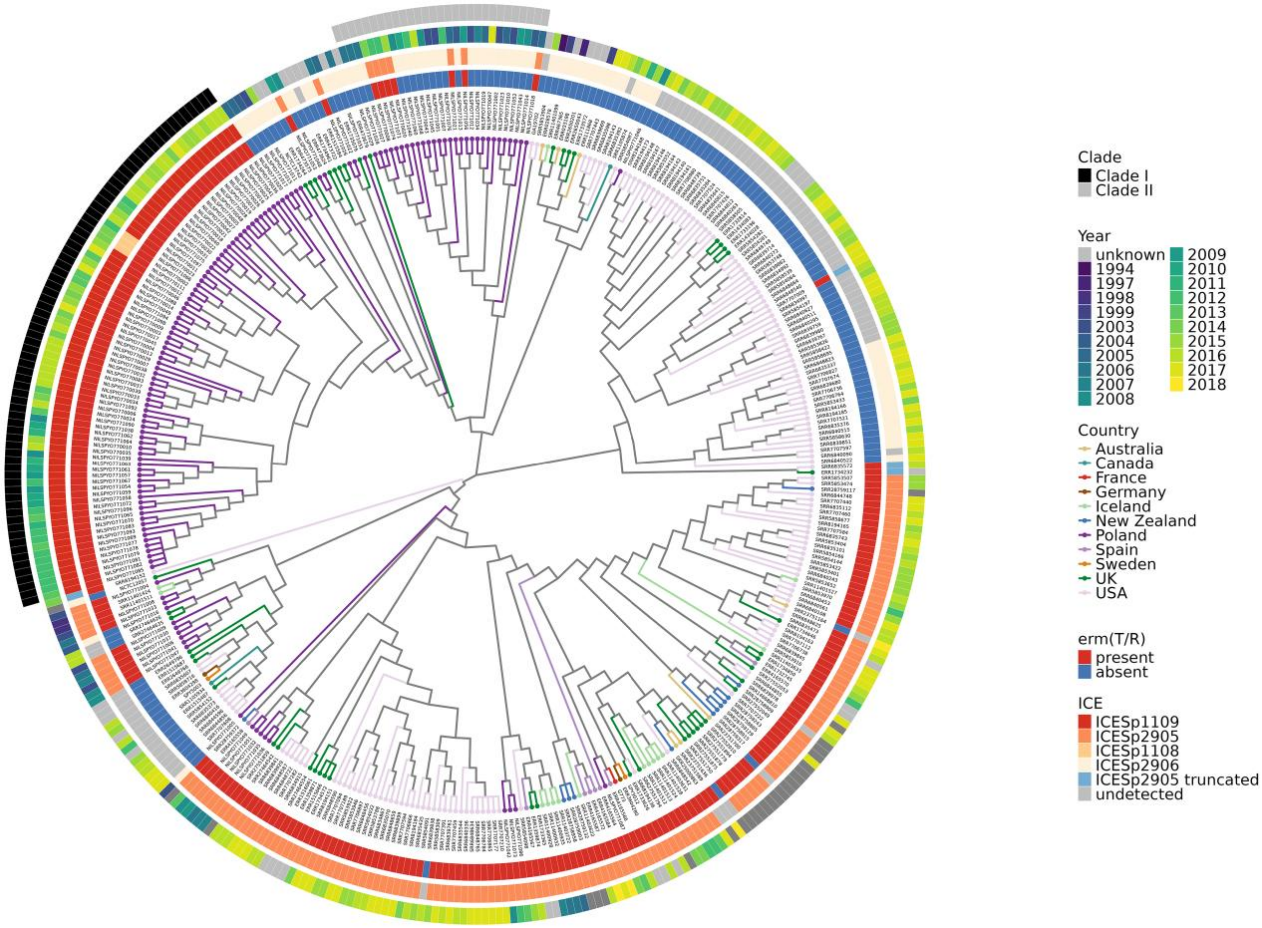
The maximum-likelihood core single-nucleotide polymorphism phylogenetic tree of 389 *Streptococcus pyogenes* M77/ST63 isolates including 136 Polish isolates, and strains from other countries (BioProjects accession numbers indicated in Supplementary File 3). The innermost ring denotes *erm*(TR) presence or absence; the second ring indicates the relevant integrative and conjugative element presence in the strain; and in the third ring, the year of sample isolation is presented. The clades representing 2 major groups of Polish isolates analyzed in this study are marked in the outermost ring. The NILSPYO771001 genome was used as a reference sequence.

Most M77 strains isolated in Poland and worldwide (>60% according to the PubMLST database (https://pubmlst.org/organisms?title=Streptococcus+pyogenes) are classified as ST63. NILSPYO771001, the first complete genome of the erythromycin-sensitive Polish M77 *S. pyogenes* strain, was used as a reference genome for the SNP tree construction. The strain was isolated in 2003, that is, earlier than the NCTC13742 type strain, which was collected in 2015. The genomes of the Polish M77*/*ST63 isolates (n = 136) were compared to M77/ST63 isolates (n = 253) from the UK, Iceland, Spain, Germany, France, Sweden, Canada, Australia, New Zealand, and the US, whose genomes were available in public databases (accessed on 1 March 2024) (Figure 5) [10, 25, 26]. A total of 1344 core genome SNPs were identified in the tested dataset that included strains isolated worldwide. The number of polymorphic sites detected among M77 strains is higher than observed among highly clonal serotypes such as M1 or M3, where differences do not exceed 100 SNPs [39]. The Polish M77 isolates had a maximal pairwise SNP difference of 76 (average difference of 22 core SNPs), and when all M77 isolates were included, the maximal pairwise SNP difference increased to 87 (average difference of 38 core SNPs) (Supplementary File 11).

The majority of Polish strains form 2 separated clusters with clonal distribution (clades) (Figure 5). One of them, clade I, groups all of the isolates containing *erm*(TR) within the ICESp*1109* hybrid element (n = 75), NILSPYO771066, NILSPYO770111, and NILSPYO770002—the 3 clones with the distantly inserted ICE*Sp2906* and *ICESp1108-*like elements in their chromosomes. The first strain (NILSPYO771038), with identified ICE*Sp1109*, was isolated in 2008, and in the 2013–2017 period this clone dominated the population of the Polish MRSP strains. In total, 487 core genome SNPs were detected among clade I strains and a maximal pairwise SNP difference of 25 was identified (Supplementary File 11). Clade II contains NILSPYO771001 reference strain and groups the majority of erythromycin-sensitive older clones (with ICE*Sp2906*), and 7 of 27 carrying *erm*(TR) within the ICE*Sp2905* variant element both in older (from 2003 to 2008) and newer (from 2008 to 2017) strains. In total, 235 core genome SNPs were identified in clade II, with a maximal pairwise SNP difference of 20.

The average pairwise difference of 10 core SNPs in clade I and 8 core SNPs in clade II (Supplementary File 11), respectively, indicate their clonal distribution.

Other Polish isolates with ICE*Sp2905* or its variant and a few carrying ICE*Sp2906* cluster predominantly with European isolates (from the UK, Spain, and Iceland) and also with several strains originated from the US, isolated in 2016 and 2017. The majority of the strains isolated in Europe (the UK, Iceland, Spain, Germany) are more diverse than those isolated in the US, which are clonally distributed.

There were no differences between clusters identified by core SNP analysis with strains NILSPYO771001 or NCTC13742 as a reference (Supplementary Figure 4). With *S. pyogenes* NCTC13742 as a reference, 1339 core genome SNPs were detected among all tested strains. All Polish isolates (n = 136) and a majority of the isolates from other countries (n = 237) carry the *tet*(O) gene within the ICE*Sp2906* element. No correlation was found between the SNP composition in analyzed strains and their ICE repertoire (Supplementary File 11).

## DISCUSSION

The *S. pyogenes* M77 isolates collected in Poland in 2003–2017 represent a single ST63, which has also been reported in other European countries [8, 10, 25–28], and North America, Australia, and New Zealand [28]. Our phylogenetic analysis of M77/ST63 strains shows relationships between strains isolated in different parts of the globe, suggesting clonal dissemination of analyzed strains. Unfortunately, our observations are limited due to the lack of WGS data from other European countries, especially from Central Europe. In public databases, there were no data from Poland's neighboring countries, such as the Czech Republic, Slovakia, or the eastern part of Europe, and the limited dataset was available from Germany (2 isolates).

The erythromycin-resistant Polish M77/ST63 isolates carry the *erm*(TR) gene within the *tet*(O)-containing ICE elements. In the early isolates *erm*(TR) is found in the already known ICE*Sp2905* [6] or its variant. Starting from 2008 it is located in ICE*Sp1109*, the novel hybrid element described in this work, composed of the ICE*Sp2906* variant and ICE*Sp1108*-like elements. Although most M77/ST63 strains were analyzed as draft genomic sequences, the genomic organization of MGEs was confirmed by assembling the complete genomes of selected isolates using long-read sequencing. No isolates with *erm*(TR) without *tet*(O) were detected, suggesting that ICE*Sp2906* acquisition was a prerequisite for *erm*(TR) gaining. The strains carrying the ICE*Sp1109* element disseminated clonally in Poland and dominated the population of *S. pyogenes* M77/ST63. Due to the lack of sequence data from neighboring countries, we cannot determine whether such a clone is disseminated elsewhere.

The surveillance and monitoring of macrolide-resistant *S. pyogenes* have to be ongoing due to the public health challenges underlined recently also by the World Health Organization in 2024, which added these bacteria to the medium-priority group of pathogens [40].

## Supplementary Data

Supplementary materials are available at *The Journal of Infectious Diseases* online (http://jid.oxfordjournals.org/). Supplementary materials consist of data provided by the author that are published to benefit the reader. The posted materials are not copyedited. The contents of all supplementary data are the sole responsibility of the authors. Questions or messages regarding errors should be addressed to the author.

## Supplementary Material

jiae473_Supplementary_Data

## Notes

***Author contributions*.** J. G. performed the research, analyzed the data, and wrote and revised the manuscript. K. Ż., M. O. S., M. K., I. W.-P., and A. K. performed the research. S. B. B. provided analytical tools. R. G. analyzed the data. J. M. M. provided funding and analytical tools. I. S. conceived the project, provided funding, performed the research, analyzed the data, and wrote the manuscript. I. K.-Z. supervised the project, analyzed the data, and wrote and revised the manuscript.

***Ethics compliance*.** The study was conducted as continuous surveillance following the World Health Medical Association 1966 Declaration of Helsinki and the European Union rules of Good Clinical Practice.

***Financial support.*** The study was funded by the National Science Centre, Poland (grant number 2017/27/B/NZ7/00040); and by the Fondren Foundation. I. S. is a recipient of the Senior Fulbright Award.
