# Supplementary material for: ICESp1109, a Novel Hybrid Integrative Conjugative Element of Macrolide-Resistant Streptococcus pyogenes Serotype M77 Collected Between 2003 and 2017 in Poland
Source: J Infect Dis. 2024 Oct 12;231(3):e521–30. doi: 10.1093/infdis/jiae473 (PMC11911784; doi:10.1093/infdis/jiae473)
Supplement: jiae473_Supplementary_Data [file jiae473_supplementary_data.zip › M77SupplementalFileS2_FiguresS1-S4.docx]

**Supplemental File S2** Detailed description of whole-genome sequencing and complete genome assembly methods.

**Whole-genome sequencing (WGS).** Genomic DNA was purified with a Genomic Mini kit (A&A Biotechnology, Poland) and the SDS/Phenol method (14) for short-read for long-read sequencing respectively (1). DNA quality was controlled by measuring the absorbance at 260/230 using a PicoDrop instrument (Picodrop Ltd, Cambridge UK). DNA concentration was determined using the Qubit fluorimeter (Thermo Fisher Scientific, Waltham, USA) and integrity was analyzed by 0.8% agarose (Bio Standard, Prona Agarose, Burgos, Spain) gel electrophoresis. DNA libraries were constructed using Nextera XT (Illumina Inc., USA) and NEB Ultra II FS kits (NEB, USA), followed by Illumina sequencing targeting at least 50x genome coverage using the MiSeq and NextSeq 550 instruments (Illumina Inc.). Sequence quality metrics were assessed using FastQC v.0.11. (<http://www.bioinformatics.babraham.ac.uk/projects/fastqc/>) and SeqKit v.2.8.1 (<https://bioinf.shenwei.me/seqkit/>). After quality check raw sequencing reads were trimmed for quality and residual library adaptors were removed using fastp v.0.23.4 (<https://github.com/OpenGene/fastp>). Cleaned short reads were checked for contamination using Kraken2 v.2.0.8 (<https://ccb.jhu.edu/software/kraken2/>). Illumina reads were then assembled into contigs using SPAdes v.3.15.5 (<https://github.com/ablab/spades>) and Unicycler v.0.4.8 (<https://github.com/rrwick/Unicycler>) to obtain high-quality draft genomes. To facilitate later analysis for mobile genetic elements (MGEs) detection in fragmented assemblies, draft genomes were scaffolded by the RagTag v.2.1.0 tool (2) with *S. pyogenes* NCTC13742 (Acc. No. LS483386.1) as the reference genome. Additionally, eight representative strains were selected for the genome finishing stage to obtain complete physical maps and confirm the structure of mobile genetic elements carrying antibiotic resistance genes. Before long-read library preparation, genomic DNA was size-selected using a Short Read Eliminator kit (Circulomics, USA). Recovered DNA was used for library construction with Native Barcoding kit V14 (EXP-NBD114.24; Oxford Nanopore Technologies, Oxford, UK). The final library (0.55 µg) was loaded into the R10.4.1 PromethION flowcell and sequenced on the P2 Solo sequencer (Oxford Nanopore Technologies). Raw nanopore data was basecalled using Bonito basecaller v.0.7.3 (https://github.com/nanoporetech/bonito, Oxford Nanopore Technologies) in super accuracy mode (SUP, Qscore >10). After residual adapter removal using Porechop_ABI v.0.5.0 ([https://github.com/bonsai-team/Porechop_ABI](http://h/)) and quality filtering using NanoFilt v.2.8.0 (<https://github.com/wdecoster/nanofilt>) the dataset was quality-checked using NanoPlot v.1.42.0 (3).

**Complete genome assembly.** Long-read assembly was performed using the Trycycler v.0.5.3 pipeline (4). In brief, nanopore reads were initially assembled using: Flye v2.9 (<https://github.com/fenderglass/Flye>), Unicycler v.0.4.8 (<https://github.com/rrwick/Unicycler>), Raven v.1.8.1 (<https://github.com/lbcb-sci/raven>), and Miniasm v.0.3-r179 (<https://github.com/lh3/miniasm>). Long-read assembly was polished with medaka v1.7.2 (https://github.com/nanoporetech/medaka, Oxford Nanopore Technologies) and further with short reads using Polypolish v.0.6.0 (<https://github.com/rrwick/Polypolish>) and POLCA v.4.0.5. (<https://github.com/alekseyzimin/masurca>) tools. All tools were run with default parameters. The remaining ambiguities in the genome assembly were verified by the PCR amplification of DNA fragments (The list of PCR primer sequences and PCR conditions for validation of the genomic regions are listed in Table 1 below), followed by Sanger sequencing with an ABI3730xl Genetic Analyzer (Thermo Fisher Scientific) using BigDye Terminator Mix v.3.1 (Thermo Fisher Scientific). All possible sequence errors and misassemblies were manually corrected using Seqman v.9.1 software (https://www.dnastar.com/software/lasergene/, DNAStar, Madison, WI, USA). Final assemblies were quality-checked using Quast v.5.0.2 (<https://quast.sourceforge.net/>). Whole genome alignments were constructed using Mummer4 (<https://mummer4.github.io/>) and pyGenomeViz v.0.4.4 (<https://github.com/moshi4/pyGenomeViz>).

**The list of PCR primers used for complete genome validation.**

**Table 1.** List of PCR primers used for PCR amplification and Sanger sequencing of genomic regions validated during the genome finishing stage.

| **Sample names** | **Primer name** | **Primer sequence** | **Annealing temperature** |
| --- | --- | --- | --- |
| NILSPYO771001 | 771001_600F | ACGATTGTCTATCAGCGTTC | 52°C |
|  | 771001_600R | ACTCGAGGCTAATCTCTTTG | 52°C |
|  | 771001_1774F | GCGTTTGGTTAGCTTCTTTG | 52°C |
|  | 771001_1775R | AGGCCCTAAAGGTGAAGAC | 52°C |
| NILSPYO771039 | 771039_832F | CGCCGGAAAAGATGGTAAAG | 52°C |
|  | 771039_832R | CTGCTGTAAAGAATGGATTGG | 52°C |
|  | 771039_1021F | TGTTAGCATAAAGCACCTCC | 52°C |
|  | 771039_1021R | AGGTTATCCAACTCACCAAG | 52°C |

The genomic fragments were amplified in 20-µL reaction mixtures containing 2 μL (ca. 10 ng) of template DNA, 0.1 μL (5 U/μL) of OptiTaq DNA polymerase (EURx, Gdańsk Poland), 2 μL of 10x Pol buffer C with MgCl2 (1.5 mM), 0.8 μL of dNTPs (0.2 mM each), and 0.4 μL (0.2 µM) of region-specific primers (Table 1). The PCR conditions were: 3 min of initial denaturation at 95oC, followed by 35 cycles of 15 s at 95°C, 15 s at 52°C, 30 s at 72°C, and a final extension period of 5 min at 72°C. The amplified products were visualized by agarose gel electrophoresis (1%, wt/v) and ethidium bromide staining. The amplicons were purified using EPPiC Fast kit (A&A Biotechnology, Gdańsk, Poland) and directly sequenced with the same primers used for PCR amplification.


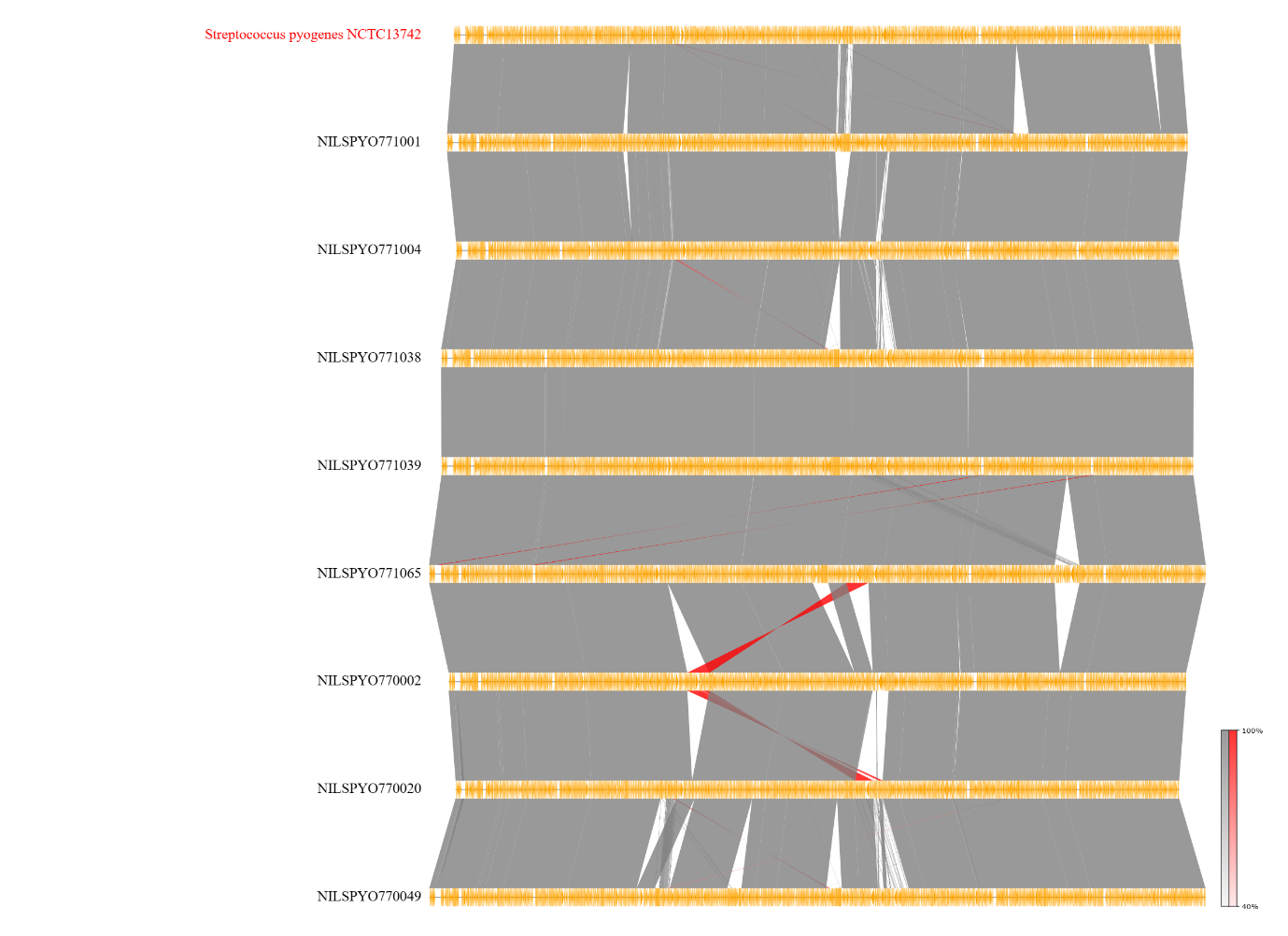


**Supplemental Figure S1** Whole genome alignment of *S. pyogenes* complete genomes obtained in this study to *S. pyogenes* NCTC13742 strain. Polish *S. pyogenes* strains are aligned to the reference sequence from the first strain in the Polish dataset NILSPYO771001 isolated in 2003 (top) to the last strain analyzed NILSPYO770049 isolated in 2017 (bottom).


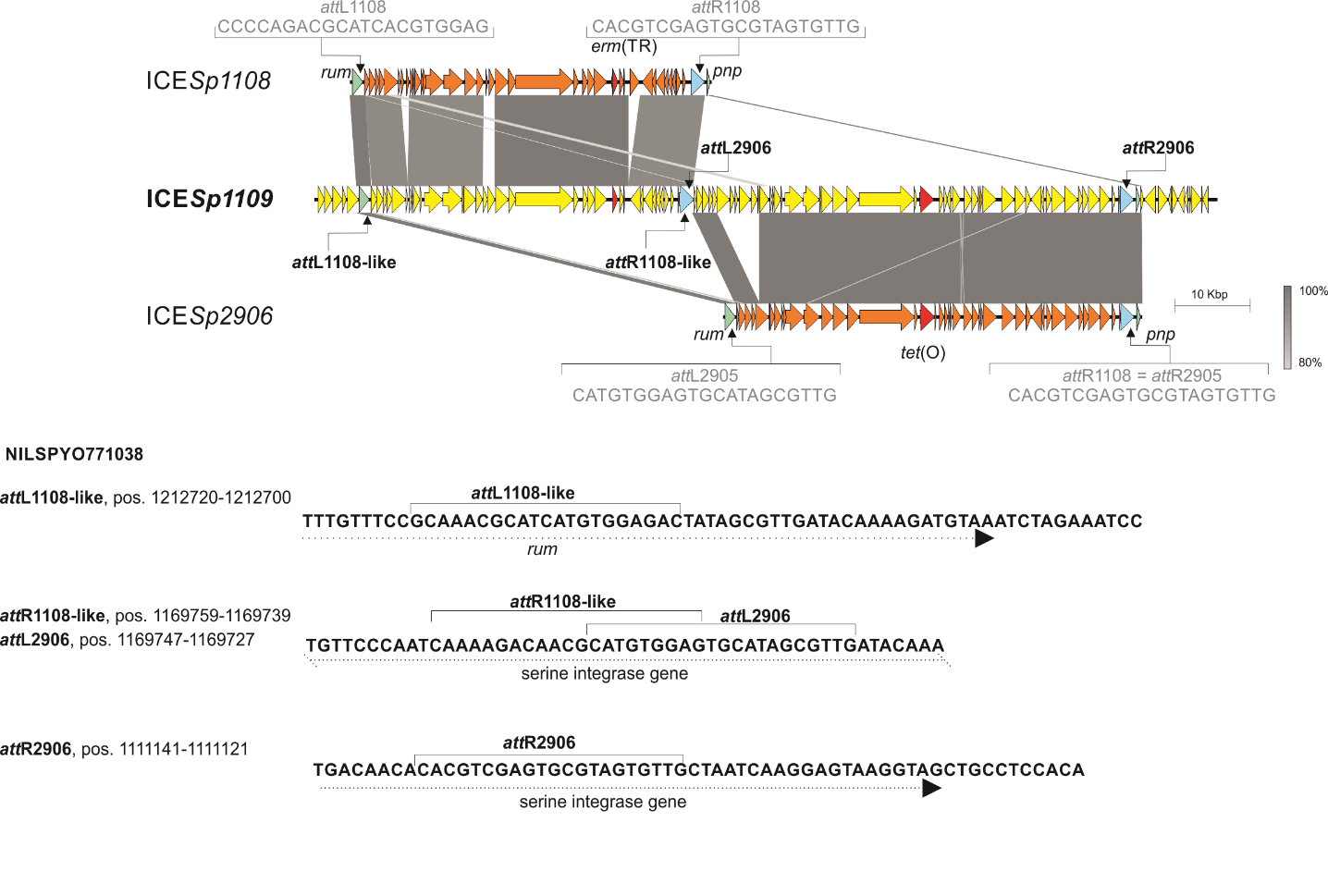


**Supplemental Figure S2** Schematic representation of ICE*Sp1109* with detected integration sites.

For comparison, ICE*Sp1108* (GenBank Acc. No. U09422.1) and ICE*Sp2906* (GenBank Acc. No. U09422.1 and HE575245.1 assembled manually) are presented. The *rum* and *pnp* genes are marked in light green, the serine integrase gene is marked in blue. The sequences *att*L and *att*R are shown according to Brenciani *et al*. (5), of ICE*Sp1108* - *att*L1108 and *att*R1108, of ICES*p2905* *att*L2905 and *att*R2905. Bolded – putative *att*L and *att*R sequences detected in ICE*Sp1109.*

Below, the positions of the *att*L and *att*R sequences are shown in the NILSPYO771038 genome. The *att* nucleotide sequence is marked with the bracket. The lines below the nucleotide sequences indicate *orf*s. The arrowheads show the last nucleotide of the stop codon.


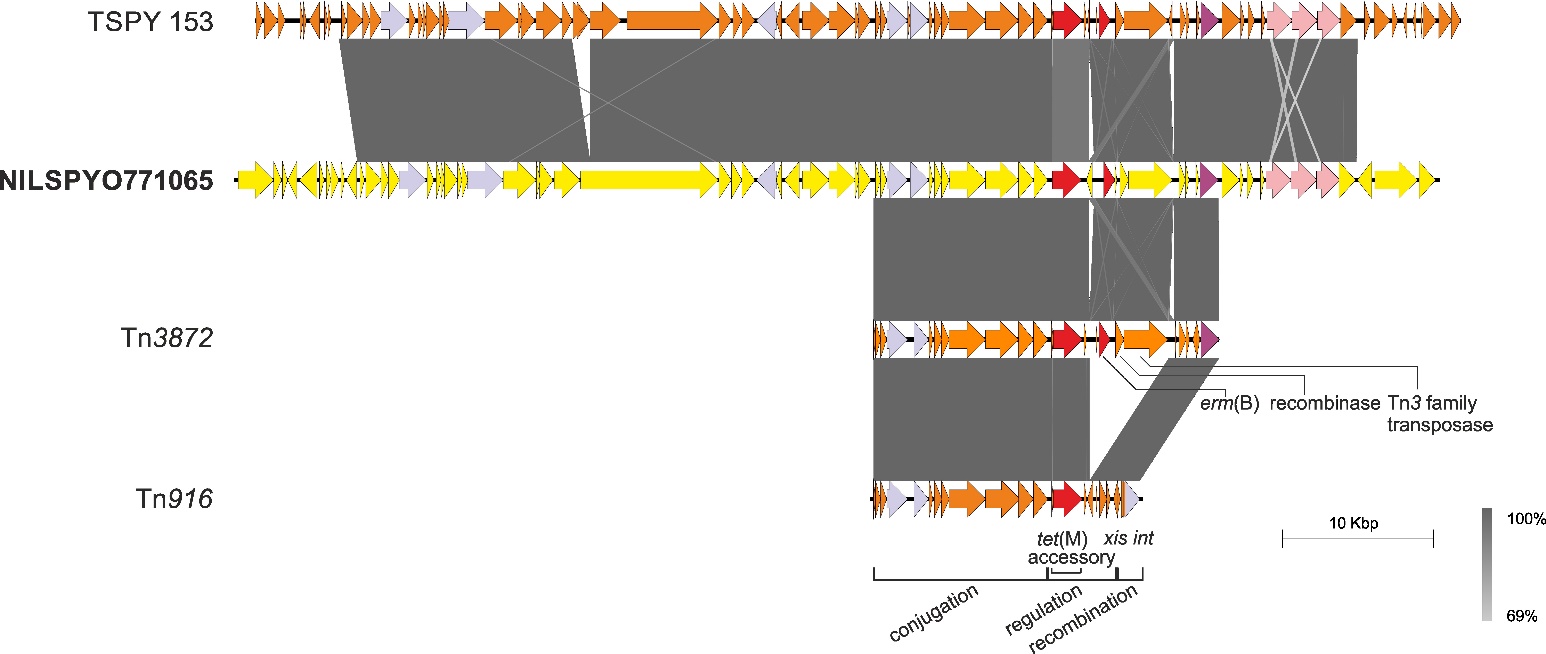


**Supplemental Figure S3** Schematic representation of the NILSPYO771065 genome region carrying Tn*3872.*

The arrows indicate genes. In red- antibiotic resistance genes, in blue – recombinase genes – light-serine recombinase, dark – tyrosine recombinase, in violet - genes detected by ICEscreen (https://icescreen.migale.inrae.fr/) as related to conjugative transfer: relaxase, VirB4, and coupling protein. TSPY153 – *S. pyogenes* strain TSPY153 (GenBank Acc. No. CP060639.1), Tn*3872* – *S. agalactiae* strain PHEGBS0450 transposon Tn3872 (GenBank Acc. No. OP715845.1), Tn*916* - *Enterococcus faecalis* DS16 transposon Tn916 (GenBank Acc. No. U09422.1).


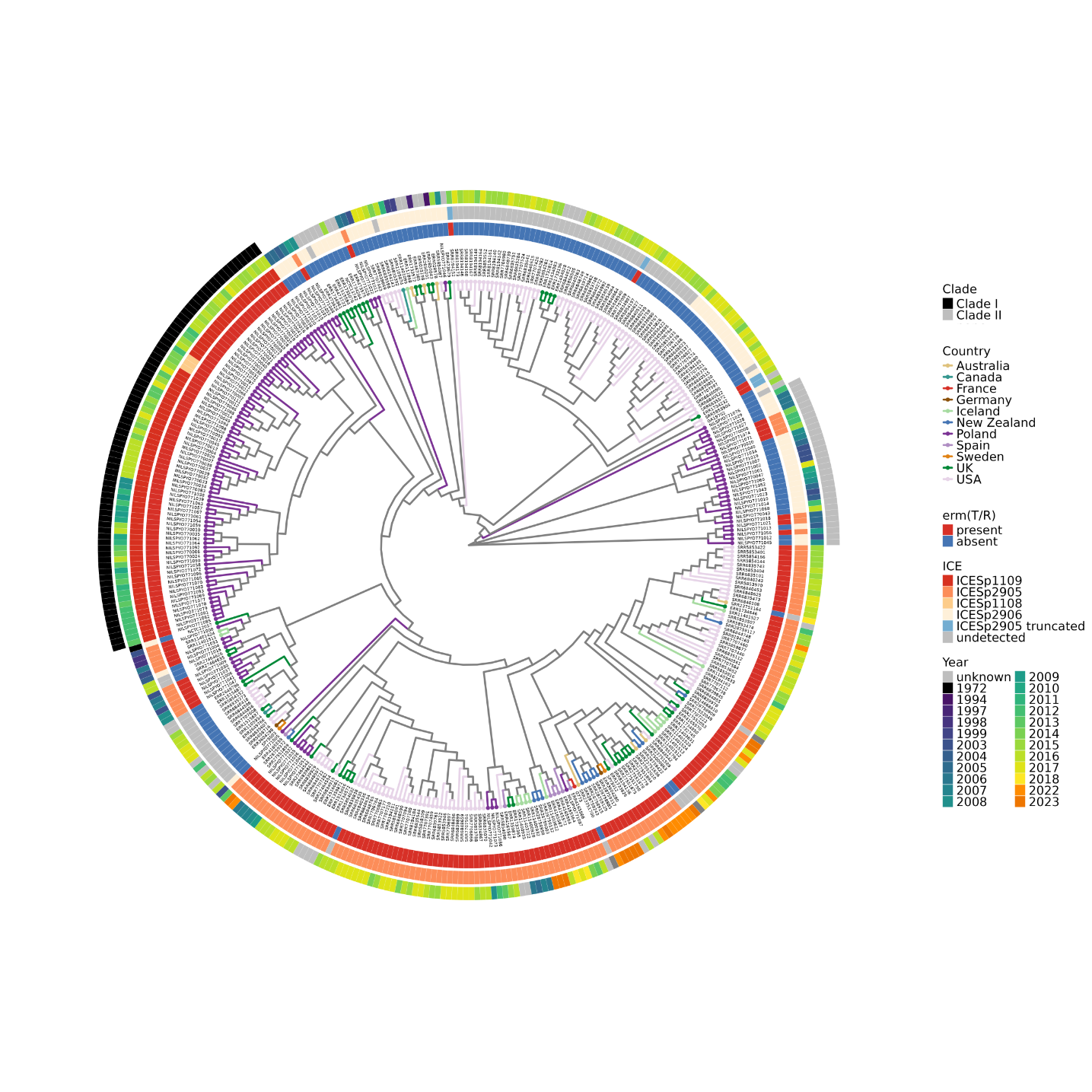


**Supplemental Figure S4** The core-SNP phylogenetic tree of 389 *S. pyogenes* M77/ST63 isolates including 136 Polish isolates, and strains from other countries (BioProjects accession numbers indicated in the Supplemental File S2). The innermost ring denotes *erm(TR)-*presence or -absence, the second ring – the relevant ICE element presence in the strain, and in the third ring, the year of sample isolation is presented. The clades representing two major groups of Polish isolates analyzed in this study are marked in the outermost ring. The *S. pyogenes* NCTC13742 genome (Acc. No. LS483386.1) was used as a reference sequence.

**Supplemental references**

1. Wilson K. Preparation of genomic DNA from bacteria. In: F M Ausubel, R Bent, R E Kingston et al (eds), J Wiley & Sons, New York, Pp Current protocols in molecular biology [Internet]. Curr Protoc Mol Biol; 1987 [cited 2023 Dec 14]. p. 2.10–2.12. Available from: https://pubmed.ncbi.nlm.nih.gov/18265184/

2. Alonge M, Lebeigle L, Kirsche M, Jenike K, Ou S, Aganezov S, et al. Automated assembly scaffolding using RagTag elevates a new tomato system for high-throughput genome editing. Genome Biol [Internet]. 2022 Dec 1 [cited 2024 Apr 30];23(1):1–19. Available from: https://genomebiology.biomedcentral.com/articles/10.1186/s13059-022-02823-7

3. De Coster W, D’Hert S, Schultz DT, Cruts M, Van Broeckhoven C. NanoPack: Visualizing and processing long-read sequencing data. Bioinformatics [Internet]. 2018 Aug 1 [cited 2024 Apr 30];34(15):2666–9. Available from: https://dx.doi.org/10.1093/bioinformatics/bty149

4. Wick RR, Judd LM, Cerdeira LT, Hawkey J, Méric G, Vezina B, et al. Trycycler: consensus long-read assemblies for bacterial genomes. Genome Biol [Internet]. 2021 Dec 1 [cited 2024 Apr 30];22(1):1–17. Available from: https://genomebiology.biomedcentral.com/articles/10.1186/s13059-021-02483-z

5. Brenciani A, Tiberi E, Bacciaglia A, Petrelli D, Varaldo PE, Giovanetti E. Two distinct genetic elements are responsible for erm(TR)-mediated erythromycin resistance in tetracycline-susceptible and tetracycline-resistant strains of Streptococcus pyogenes. Antimicrob Agents Chemother [Internet]. 2011 May [cited 2022 Aug 25];55(5):2106–12. Available from: https://pubmed.ncbi.nlm.nih.gov/21343455/
