# Supplementary material for: ICESp1109, a Novel Hybrid Integrative Conjugative Element of Macrolide-Resistant Streptococcus pyogenes Serotype M77 Collected Between 2003 and 2017 in Poland
Source: J Infect Dis. 2024 Oct 12;231(3):e521–30. doi: 10.1093/infdis/jiae473 (PMC11911784; doi:10.1093/infdis/jiae473)
Supplement: jiae473_Supplementary_Data [file jiae473_supplementary_data.zip › supplemental material legends.docx]

**Supplemental information**

**Supplemental File S1** Polish *S. pyogenes* M77 strains description.

**Supplemental File S2** Detailed description of whole-genome sequencing and complete genome assembly methods.

**Supplemental File S3** List of BioProjects used in phylogenetic analysis.

**Supplemental File S4** Virulence factor genes identified in the Polish *S. pyogenes* M77 genomes.

**Supplemental File S5** Insertion sequences identified in the Polish *S. pyogenes* M77 genomes.

**Supplemental File S6** Integrative and Conjugative Elements identified in the Polish *S. pyogenes* M77 genomes.

**Supplemental File S7** Prophages detected in the Polish *S. pyogenes* M77 genomes.

**Supplemental File S8** Prophages homologs identified in the Polish *S. pyogenes* M77 genomes.

**Supplemental File S9** Comparison of ICE*Sp1109* sequences to ICE*Sp2905* and ICE*Sp1108.* A/ BLASTn analysis results for the ICE*Sp1109* element. B/ ICEscreen tool analysis results (detected Signature Proteins).

**Supplemental File S10** ICEscreen tool analysis results in NILSPYO771065 - detected Signature Proteins with Mobile Elements.

**Supplemental File S11** *S. pyogenes* M77 pairwise SNP distance matrices.

**Supplemental Figure S1** Whole genome alignment of complete genomes of *S. pyogenes* obtained in this study to *S. pyogenes* NCTC13742 strain.

**Supplemental Figure S2** Schematic representation of ICE*Sp1109* with detected integration sites.

**Supplemental Figure S3** Schematic representation of the NILSPYO771065 genome region carrying Tn*3872.*

**Supplemental Figure S4** The maximum-likelihood core-SNP phylogenetic tree of 389 *S. pyogenes* M77/ST63 isolates including 136 Polish isolates, and strains from other countries, with *S. pyog*enes NCTC13742 genome as a reference sequence.
